# *Tripogon loliiformis* tolerates rapid desiccation after metabolic and transcriptional priming during initial drying

**DOI:** 10.1038/s41598-023-47456-3

**Published:** 2023-11-23

**Authors:** Pauline A. Okemo, Isaac Njaci, Young-Mo Kim, Ryan S. McClure, Matthew J. Peterson, Alexander S. Beliaev, Kim K. Hixson, Sagadevan Mundree, Brett Williams

**Affiliations:** 1 School of Biology and Environmental Science, Queensland University of Technology, Brisbane, QLD Australia; 2 Centre for Agriculture and the Bioeconomy, Queensland University of Technology, Brisbane, QLD Australia; 3 Queensland Alliance for Agriculture and Food Innovation, University of Queensland, Brisbane, QLD Australia; 4 Earth and Biological Sciences Directorate, Pacific Northwest National Laboratory, Richland, WA USA; 5 Physical and Chemical Sciences Directorate, Pacific Northwest National Laboratory, Richland, WA USA; 6 Biosphere 2, The University of Arizona, Oracle, AZ USA

**Keywords:** Proteomics, Transcriptomics, Plant molecular biology, Plant physiology, Plant stress responses

## Abstract

Crop plants and undomesticated resilient species employ different strategies to regulate their energy resources and growth. Most crop species are sensitive to stress and prioritise rapid growth to maximise yield or biomass production. In contrast, resilient plants grow slowly, are small, and allocate their resources for survival in challenging environments. One small group of plants, termed resurrection plants, survive desiccation of their vegetative tissue and regain full metabolic activity upon watering. However, the precise molecular mechanisms underlying this extreme tolerance remain unknown. In this study, we employed a transcriptomics and metabolomics approach, to investigate the mechanisms of desiccation tolerance in *Tripogon loliiformis*, a modified desiccation-tolerant plant, that survives gradual but not rapid drying. We show that *T. loliiformis* can survive rapid desiccation if it is gradually dried to 60% relative water content (RWC). Furthermore, the gene expression data showed that *T. loliiformis* is genetically predisposed for desiccation in the hydrated state, as evidenced by the accumulation of MYB, NAC, bZIP, WRKY transcription factors along with the phytohormones, abscisic acid, salicylic acid, amino acids (e.g., proline) and TCA cycle sugars during initial drying. Through network analysis of co-expressed genes, we observed differential responses to desiccation between *T. loliiformis* shoots and roots. Dehydrating shoots displayed global transcriptional changes across broad functional categories, although no enrichment was observed during drying. In contrast, dehydrating roots showed distinct network changes with the most significant differences occurring at 40% RWC. The cumulative effects of the early stress responses may indicate the minimum requirements of desiccation tolerance and enable *T. loliiformis* to survive rapid drying. These findings potentially hold promise for identifying biotechnological solutions aimed at developing drought-tolerant crops without growth and yield penalties.

## Introduction

Most flowering angiosperms can withstand up to 60% water loss. Further decreases in the water potential result in compromised growth and development and eventually cell death. However, a distinct group of plants has evolved a remarkable capacity to survive desiccation, where their vegetative tissues equilibrate with the air, remain in a quiescent state for extended periods, and regain full metabolic activity upon watering^1–4^. These plants are called desiccation tolerant and two categories of desiccation-tolerant plants are recognised: true desiccation and modified desiccation tolerant plants. True desiccation tolerance plants include algae, lichens, and mosses and can endure rapid desiccation and revive upon rehydration^5^. Their desiccation can occur within a brief time; thus, their protection mechanisms are constitutive rather than inducible^5^. Modified desiccation-tolerant plants do not have constitutive protective mechanisms and require gradual drying to activate their protective mechanisms. To survive, modified desiccation-tolerant plants employ multiple physiological and morphological mechanisms that delay water loss, prevent cellular damage, and activate protective processes that enhance tolerance^3,6^.

Considerable research has been conducted on the morphology and the biochemical mechanisms underlying desiccation tolerance in these plants^1,7–9^. Like other resurrection plants, the native Australian resurrection grass, *Tripogon loliiformis* survives desiccation and recovers within 72 h of watering. Studies have shown that *T. loliiformis* tightly regulates Programmed Cell Death (PCD) pathways, energy and nitrogen metabolism, photosynthesis, trehalose metabolism, autophagy, reactive oxygen species (ROS) scavenging systems and sugar accumulation as part of its survival strategy during desiccation^10,11^. Importantly, *T. loliiformis* dehydrating shoots demonstrate distinct responses from roots, particularly in sugar metabolism and autophagy regulation in shoots but not roots. Conversely, recent studies of *Craterostigma plantagineum* showed an opposite pattern with autophagy-related genes upregulated in roots compared with leaves during dehydration^12^.

During the drying process, cellular protection mechanisms induced by modified desiccation-tolerant angiosperms are vital for their survival, but these mechanisms require time for implementation. As a result, some of these plants cannot withstand rapid dehydration^5^. Based on physiological and molecular changes of various modified desiccation-tolerant angiosperms, researchers have proposed the boundary for the relative water content between dehydration and desiccation to be approximately 40% relative water content^13^. Sugars play a crucial role as an essential energy source for plants and accumulate earlier than other metabolites during dehydration^14^. Although sugars are not the primary players in osmotic regulation, they are used by plants to synthesize amino acids, organic acids and other metabolites that directly contribute to osmotic regulation^15^. In modified desiccation-tolerant plants, changes in sugar metabolism occur during drying to promote desiccation tolerance. For instance, true desiccation-tolerant plants like *Tortula ruraliformis* and mosses rely on sucrose for cellular protection^16^. They do not increase their soluble sugar content during dehydration but maintain high sucrose content constitutively^5^. In contrast, modified desiccation-tolerant plants accumulate considerable sucrose during dehydration^17,18^.

The modified desiccation-tolerant plant *Craterostigma plantagenium* expresses desiccation specific proteins related to late-embryogenesis abundant proteins (LEAs), proteins responsive-to the plant hormone abscisic acid (ABA), and dehydrins^6,19^. These proteins function in cellular protection, especially in seed desiccation^20,21^. Similarly, LEA proteins are expressed in the vegetative tissues of *Sporobolus stapfianus*^22^*.* Apart from water deficit, other stresses including: salt stress, cold stress and exogenous ABA can also induce expression of LEA proteins^23^. LEA proteins prevent protein denaturation by enhancing protein folding. Modified desiccation-tolerant plants also use phytohormones to regulate desiccation tolerance signalling pathways. For example, *S. stapfianus* uses ABA to activate tolerance genes during dehydration^5^. In *C. plantagineum* callus, dehydration-activated genes can be induced by exogenous ABA treatment four days before dehydration^24,25^. ABA also induces desiccation tolerance in the moss *Atrichum undulatum*^25^.

As a modified desiccation tolerant plant, *T. loliiformis* requires gradual drying to survive desiccation, however, the precise mechanisms used by *T. loliiformis* to tolerate desiccation are less understood. In this study, we used a series of drying experiments involving gradual and rapid dehydration of *T. loliiformis* plants to investigate physiologically whether there is a specific point during dehydration at which *T. loliiformis* can survive rapid drying. Previous studies have used excised leaves or relied upon multiomics data for such analyses^13^. Once we identified the physiological point at which *T. loliiformis* plants could survive rapid drying, we used transcriptomic and metabolomics studies to identify the minimal requirements for *T. loliiformis* desiccation tolerance. The knowledge from this study may be useful in determining potential pathways for the generation of drought-tolerant crops.

## Results

### *T. loliiformis* survives gradual but not rapid dehydration

**Figure 1:**
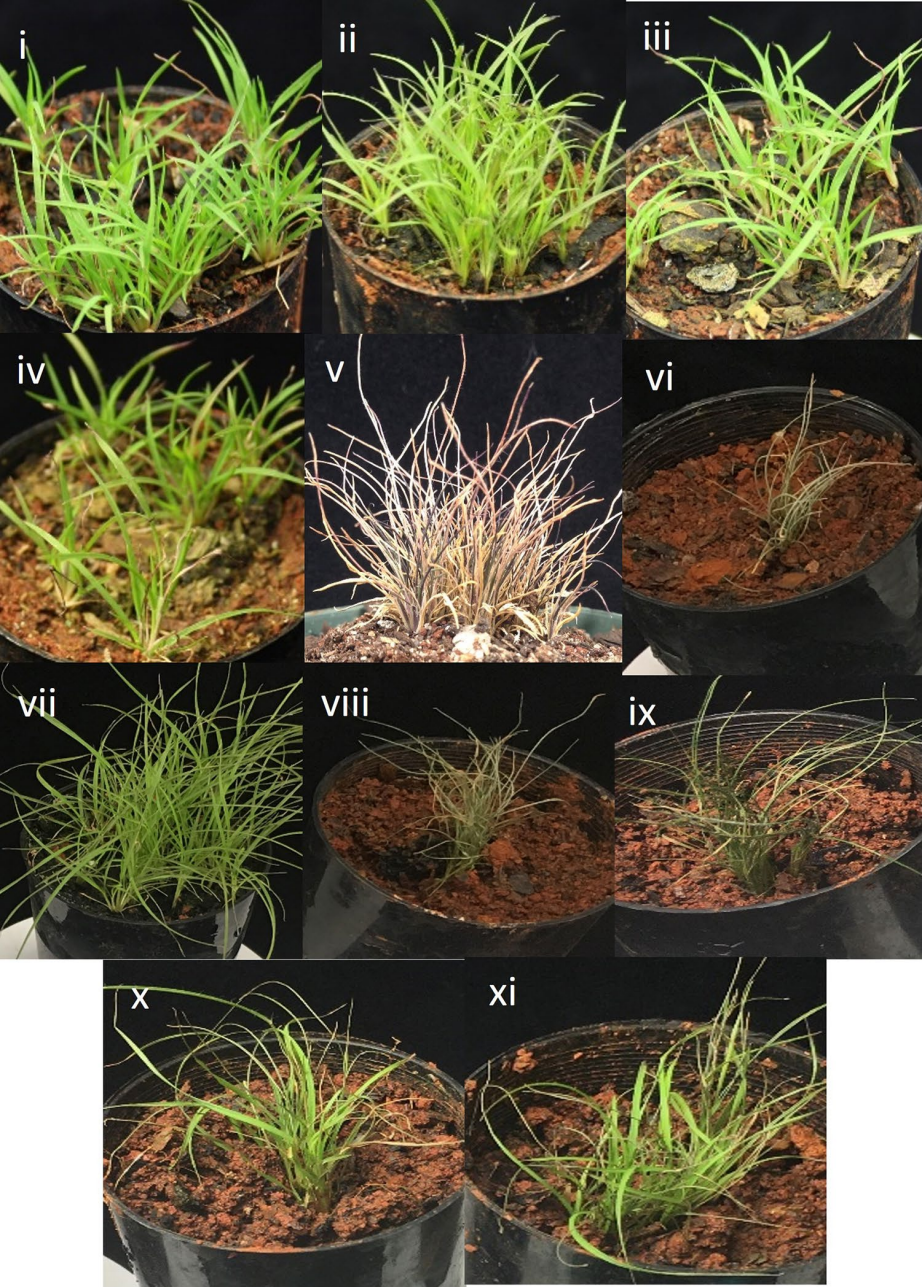
Morphological representation of *T. loliiformis* plants at different desiccation states (**i**–**v**) and rehydration after gradual and rapid desiccation (**vi**–**xi**). (**i**) Hydrated, (**ii**) 100–80 RWC (**iii**) 70–60 RWC, (**iv**) 50–40 RWC and (**v**) 10 RWC. (**vi**) Rapidly dehydrated *T. loliiformis*, (**vii**) Hydrated *T. loliiformis* before rapid dehydration, (**viii**) 100–80 RWC before rapid desiccation (**ix**–**xi**) 70–60, 50–40 and 30–20 RWC respectively before rapid desiccation. Observations made at 72 h post-watering. RWC is Relative Water Content. Ca is approximately.

True desiccation-tolerant plants possess constitutive cellular repair systems that enable them to survive rapid drying. In contrast, modified desiccation-tolerant plants require a gradual drying process to develop desiccation tolerance. To determine whether there is a specific point at which *T. loliiformis* can survive rapid dehydration, we gradually dried the plants to distinct hydration levels before rapidly drying them until desiccated. Hydrated and fully desiccated plants that were dried gradually were used as negative and positive controls, respectively. As shown in Fig. 1, hydrated and mildly dehydrated plants (100–80% RWC) did not survive rapid desiccation and did not resurrect upon watering. Plants gradually dried to ca. 60% RWC and below survived rapid desiccation and resurrected upon rehydration.

### Rapidly dehydrated *T. loliiformis* plants have a higher cell death rate compared to gradually dehydrated plants

**Figure 2:**
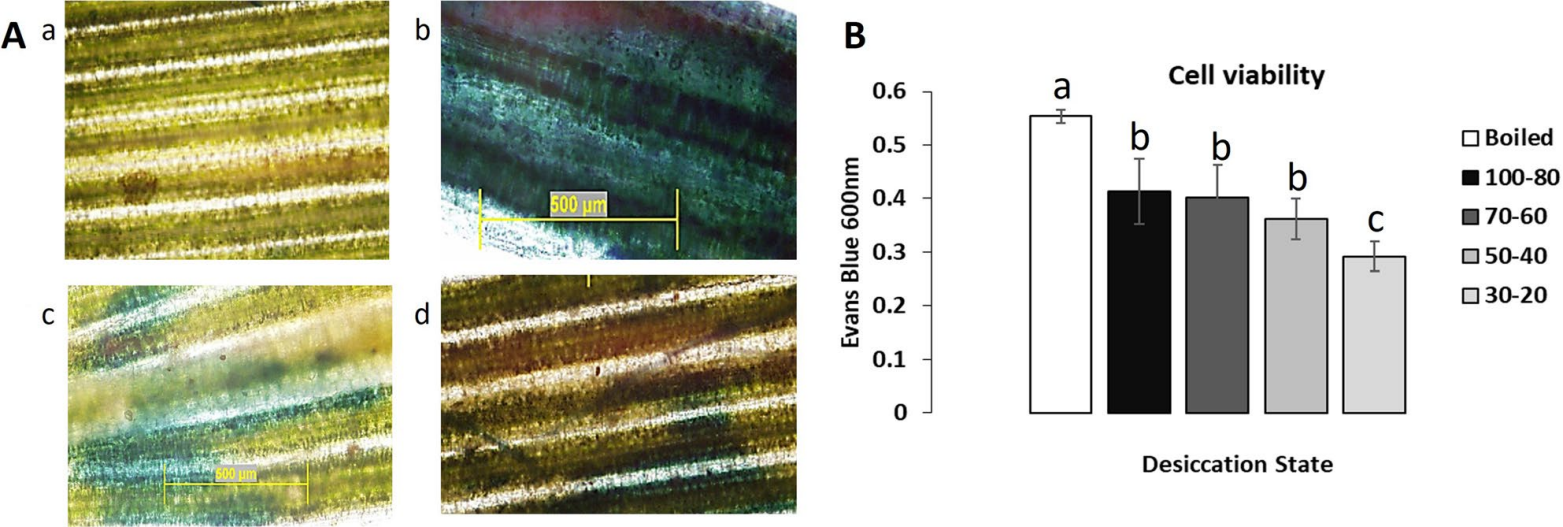
Plants gradually dehydrated to ca. 60% RWC displayed less cell death when rapidly dried. (**A**) Evans blue staining (a) Hydrated shoot without rapid dehydration, (b) 100–80 RWC after rapid dehydration, (c) 70–60 RWC after rapid dehydration, d) 30–20 RWC after rapid dehydration. (**B**) Cell viability graph. All data was recorded as means. *p* value < 0.05. RWC is Relative Water Content. Ca is approximately.

Persistent stress leads to cell injury, the loss of membrane integrity and eventually cell death. Evans Blue, an azo dye, is commonly used to assess cell vitality as it penetrates ruptured membranes and stains damaged or dead cells^26^. To examine the impact of rapid versus gradual dehydration on *T. loliiformis* cell vitality, we gradually and rapidly desiccated plants and stained the vegetative tissue with Evans Blue dye. When rapidly dried without time to acclimate, hydrated and mildly hydrated (ca. 80% RWC) plants, showed the highest levels of cell death and did not recover upon watering (Fig. 2). Conversely, plants, that were gradually dried to ca. 60% and ca. 30% RWC before rapid dehydration, resurrected upon watering and displayed lower levels of staining and cell death. This indicates that gradual acclimation (adaptation) at/to ca. 60% RWC allows the plant to withstand further drying irrespective of the speed of further drying.

### *T. loliiformis* prepares early for desiccation and recovery by accumulating necessary metabolites before 60% RWC and in rehydration

**Figure 3:**
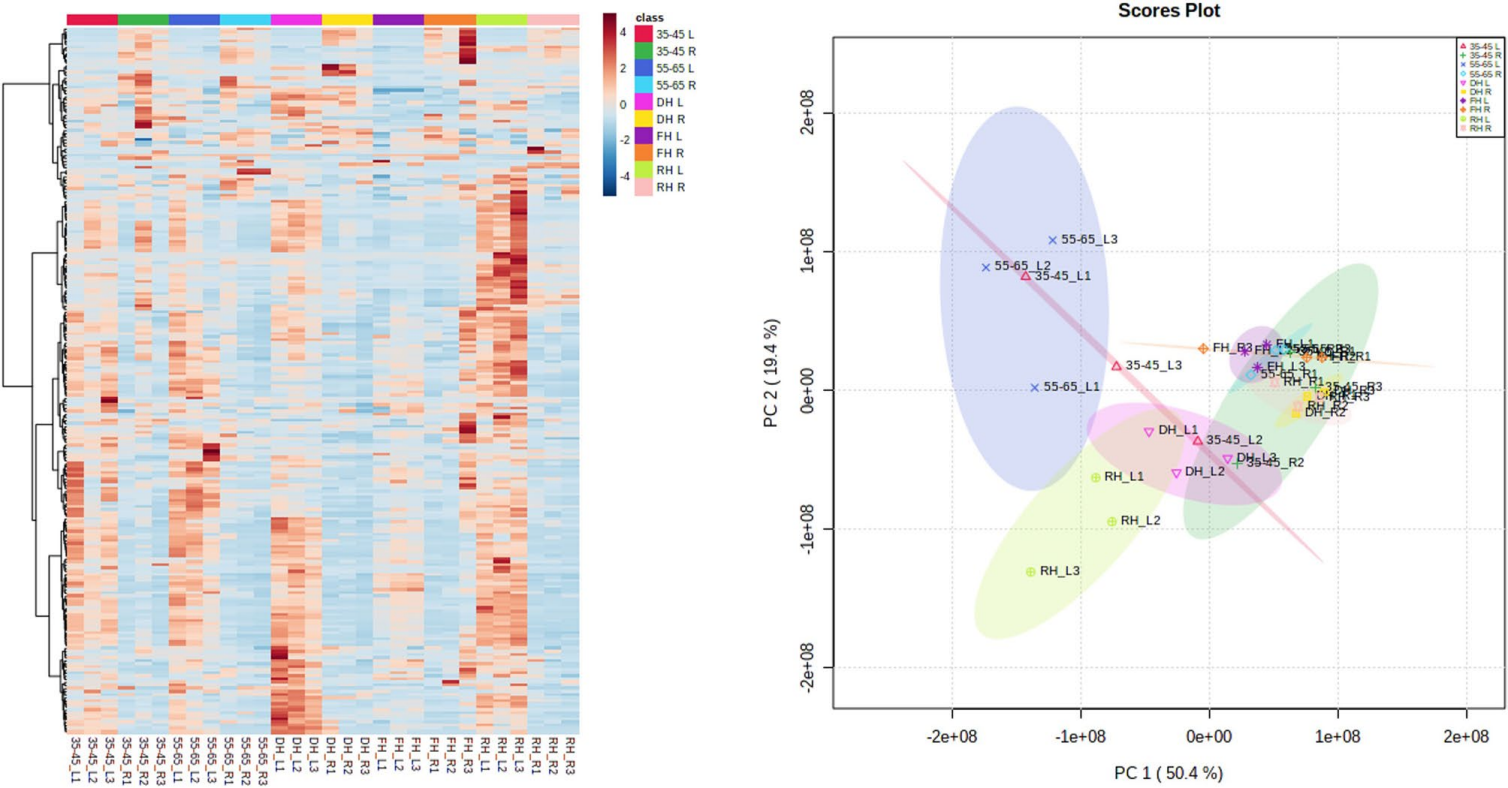
Shoots of *T. loliiformis* accumulate more metabolites than roots during dehydration. (**A**) Heatmap of different metabolites between hydrated and dehydrated shoots and roots of *T. loliiformis*. (**B**) PCA analysis of different metabolites between hydrated and dehydrated shoots and roots. *DH* Dehydrated, *RH* Rehydrated, *L* Leaf, *R* Root.

Plant development is plastic and plants have the capacity to pivot their biosynthetic and catabolic pathways towards survival rather than growth and development when exposed to abiotic and biotic stresses^27,28^. To further understand how *T. loliiformis* survives desiccation, we analysed the metabolite profiles of shoots and roots under different dehydration states. Since plants switch to alternative energy sources for survival our analysis focused on sugars, amino acids, organic acids, and various components of the TCA cycle (Table 1). Our results show higher levels of activity and metabolite expression in the shoots compared to the roots (Fig. 3), which is consistent with our previous findings showing that dehydrating *T. loliiformis* roots maintain higher levels of ATP and trehalose-6-phosphate compared to the shoots, providing protection against the adverse effects of dehydration^10^.

**Table 1:**
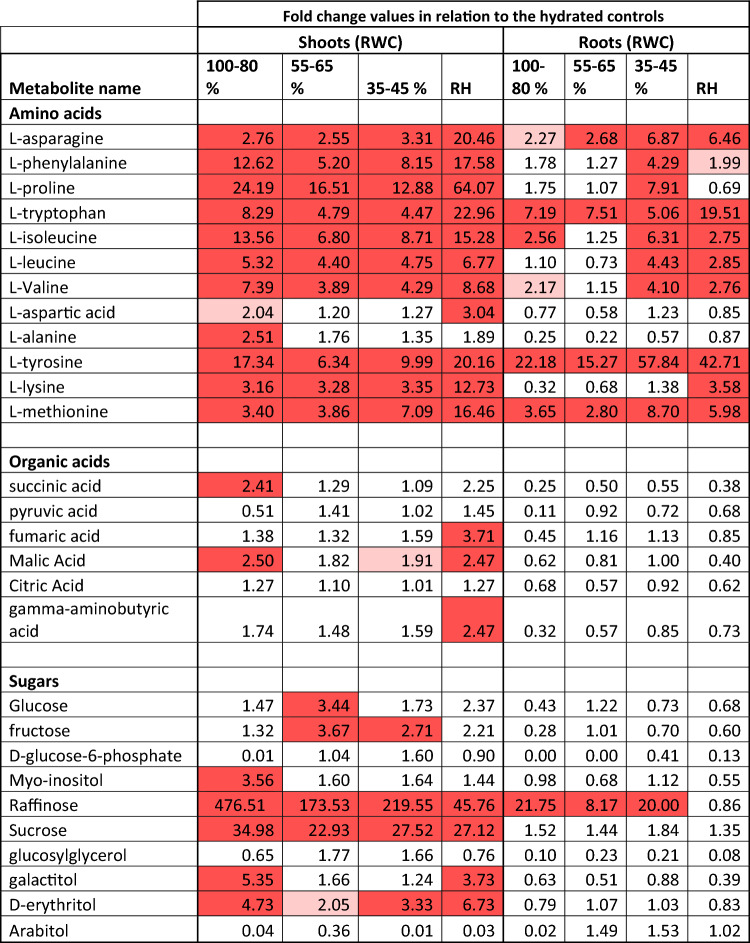
Fold change of metabolites of *T. loliiformis* shoots and roots during dehydration.

Red highlights = upregulated metabolites. *p* value < 0.05.

During the early stages of dehydration, *T. loliiformis* shoots accumulated higher amounts of myo-inositol, raffinose, sucrose, galactitol and D-erythritol compared to the hydrated shoots. Amino acids including tyrosine, isoleucine, tryptophan, phenylalanine, and proline (a key regulator of osmotic stress) also accumulated early in the shoots during dehydration. Methionine, lysine, and alanine were also accumulated during initial dehydration (Table 1). These results suggest that *T. loliiformis* shoots accumulate sugars, amino acids, and organic acids during the initial dehydration to ca. 60% RWC that aid in reprogramming the plant cells to tolerate desiccation.

In contrast, *T. loliiformis* roots displayed a distinct pattern of amino acids accumulation during severe dehydration and rehydration. Asparagine, phenylalanine, proline, tryptophan, isoleucine, leucine, valine, tyrosine and methionine accumulated only in the roots during the later stages of drying. The levels of these amino acids remained slightly elevated during rehydration compared to early and intermediate dehydration but not severe dehydration. Branched chain amino acids (BCAA) (isoleucine, valine, leucine) and aromatic amino acids (AAA) (phenylalanine, tyrosine, tryptophan) have been shown to contribute to plant stress response, provide alternative energy source for plants under long-term stress, play a role in protein biosynthesis and cell survival during abiotic and biotic stresses^29–31^. Additionally, higher methionine levels in plants have been linked with increased abiotic stress tolerance^32^. The accumulation of these amino acids in roots during prolonged dehydration coupled with our previous data on root energy status^10,33^ suggests that roots may require amino acids to survive severe dehydration and the accumulated amino acids may contribute to plant recovery.

### Upregulation of transcripts encoding cytoprotective proteins at ca. 60% RWC play a role in desiccation tolerance

**Figure 4:**
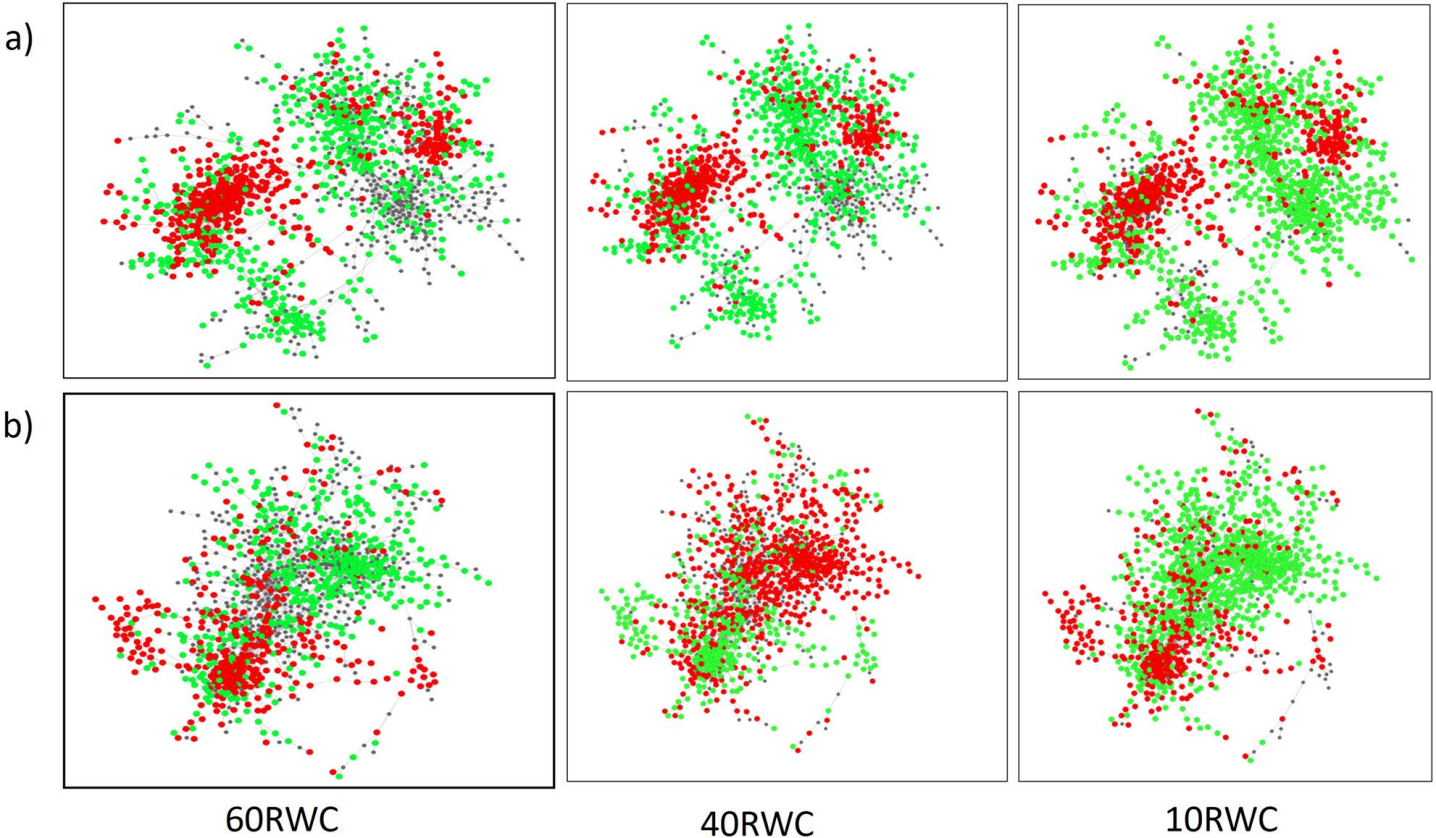
Shoots and roots of *T. loliiformis* form different networks during dehydration*.* (**a**) Shoot Network Analysis, (**b**) Root Network Analysis. Red nodes = downregulated genes while green nodes = upregulated genes. *p* < 0.05. FDR 0.05. *RWC* Relative water content.

The desiccation tolerance observed in resurrection plants is likely attributed to subtle changes in the regulatory control of genes that are present in the genomes of most plants, similar to the desiccation tolerance exhibited by seeds of flowering plants^34^. Building on the observation that gradual dehydration to ca. 60% RWC resulted in reduced cell death and faster recovery upon rehydration, we used this specific dehydration point to further investigate the molecular mechanisms used by *T. loliiformis* to tolerate desiccation. Analysis of differentially expressed genes (DEGs) showed that both shoots and roots induced expression of transcription factors (TFs) including MYB, NAC, WRKY and bZIP, carbohydrate and energy metabolism and signalling-related genes that play a role in stress tolerance (S Table 1). Notably, roots accumulated more transcripts associated with DREB genes and trehalose-6-phosphate phosphatase (catalyses the production of trehalose) while shoots contained elevated levels of transcripts encoding antioxidants and LEA (late embryogenesis abundant proteins) 2 genes. Further analysis of the DEGs in shoots and roots at 40% RWC and 10% RWC showed that T6P (trehalose 6 phosphate) transcription was upregulated at 40% RWC but downregulated at 10% RWC (S Table 2). ATG-related protein 11, a subunit that interacts with ATG1 and ATG13 to form an active complex that initiates autophagy^35^ was downregulated in roots at 10% RWC. Network analysis was conducted to further investigate gene interactions between shoots and roots at different dehydration states. Shoots displayed similar patterns across all dehydration points with key dehydration-associated genes expressed throughout dehydration (Fig. 4, S Table 2). This analysis suggests that while shoots show numerous DEGs, the overall response of this tissue is broad and does not significantly change with dehydration state. In contrast, network analysis of co-expressed genes in roots revealed distinct sections of the network representing different pathways and processes activated at different dehydration states. The response profile at 40% RWC differed markedly from both the 10% RWC or 60% RWC. Furthermore, there was also a difference in the number of DEGs at this state, with 40% RWC displaying more down-regulated genes compared to shoots at 60% RWC and < 10% RWC (Fig. 4). For example, DREB2.3 gene and, Myb-family transcription factor were downregulated in roots at 40% RWC and not 60% RWC (S Tables 1, 2).

### Increase in ABA and SA in *T. loliiformis* shoots correlates with recovery at 60% RWC

**Figure 5:**
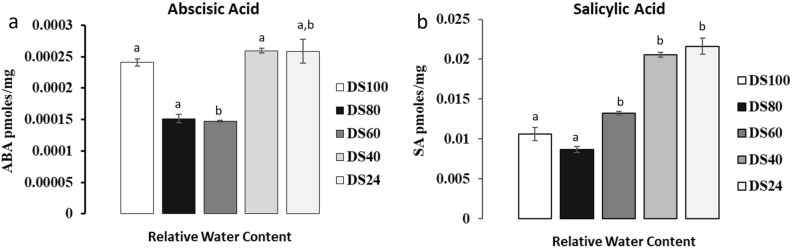
Accumulation of major stress associated phytohormone, (**a**) Abscisic Acid (ABA), (**b**) Salicylic acid (SA) in dehydrating *T. loliiformis* shoots at different desiccation states. Data is presented as means at *p* < 0.05. Samples denoted with the same letter were not significantly different from each other using a *p* value < 0.05. *DS* Dehydration state, *pmoles/mg* Picomoles/milligram, *DS24* Recovery phase.

In addition to transcriptional and metabolite responses, resurrection plants use various phytohormones, including abscisic acid, salicylic acid and jasmonic acid (JA) to support desiccation tolerance^36,37^. To determine the mechanisms that support desiccation tolerance in *T. loliiformis*, we analysed the phytohormone profile of shoots at 60% RWC and compared this profile to mildly (80% RWC) and severely (< 40% RWC) dehydrated plants. ABA induces snRK1, an important protein in energy metabolism and signalling during stress^38^. ABA concentrations were notably higher in hydrated and mildly hydrated plants compared to 60% RWC (Fig. 5a). The elevated levels of ABA present in hydrated plants suggest that *T. loliiformis* is primed to respond to desiccation in the hydrated state and can trigger signals that induce downstream responses. However, salicylic acid, another important phytohormone during drought responses accumulated at 60% RWC and continued to accumulate as dehydration progressed (Fig. 5b). These results show that phytohormones associated with drought tolerance are also present in *T. loliiformis* and salicylic acid potentially plays a vital role in desiccation tolerance.

### Decrease in jasmonate (Ja-Ile) at 60% RWC prevents cell death and plays a role in recovery

**Figure 6:**
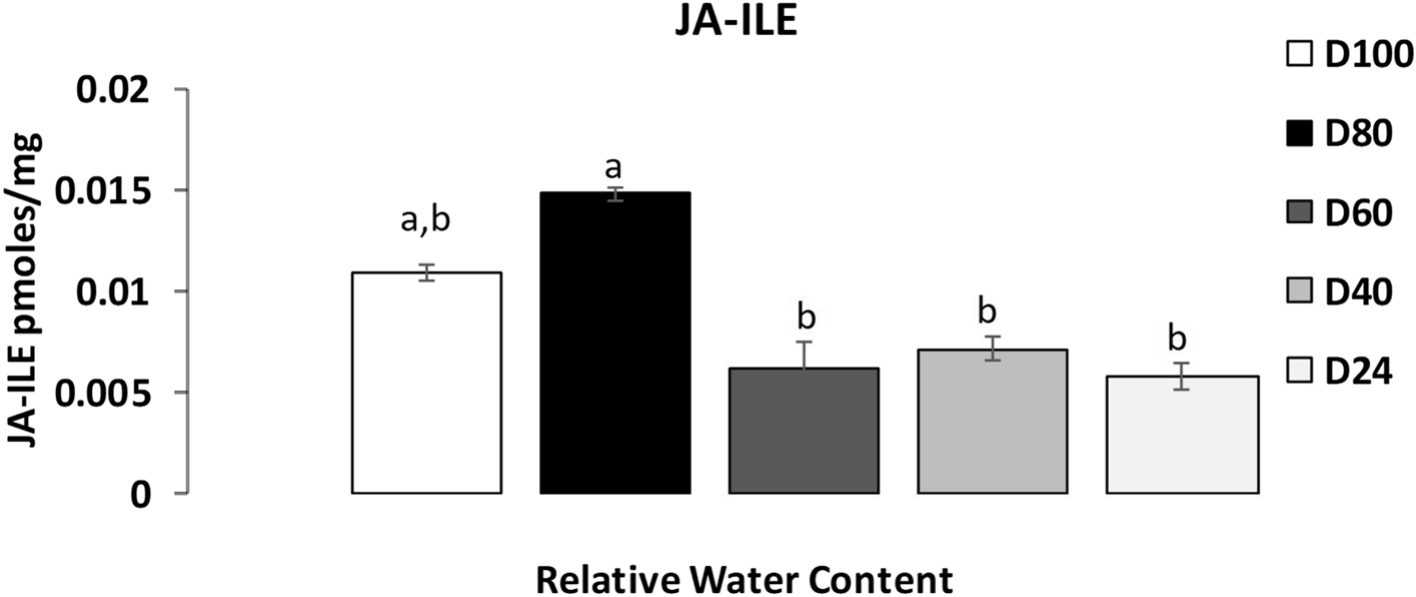
Accumulation of methyl jasmonate derivative jasmonoyl-isoleucine (JA-ILE) in dehydrating *T. loliiformis* shoots at different desiccation states. Data is presented as means at *p* < 0.05. Samples denoted with the same letter were not significantly different from each other using a *p* value < 0.05. *D* Dehydration state, *D24* Recovery state.

Jasmonates are an important class of phytohormones that play vital roles in plant defence against abiotic and biotic stresses^39,40^. However, jasmonates such as methyl jasmonate (MJ) induce apoptosis and pro-apoptotic autophagy via ROS pathways in human cells^41^. Furthermore, jasmonates induce senescence in oat plants^42^. To determine if jasmonates accumulated in *T. loliiformis* and if there were any significant differences in their accumulation pattern during dehydration, we analysed the profile of active jasmonate (JA-Ile; jasmonoyl isoleucine) of plants at 60% RWC and compared it to mildly (80% RWC) and severely (< 40% RWC) dehydrated plants. The results showed a decrease in the levels of JA-Ile as dehydration progressed with reduction beginning at 60% RWC and progressing to 40% RWC and in rehydrated plants (Fig. 6). A reduction in JA-Ile could play a role in suppressing ROS damage, apoptosis, and senescence in *T. loliiformis* during desiccation.

## Discussion

Most flowering angiosperms can survive water loss to 60% RWC. Any further decrease leads to stress and eventually cell death. Desiccation tolerant plants survive water loss to < 10% RWC and regain full metabolic capacity within 72 h of watering. Previous studies have focused on excised materials or multiomics analyses to pinpoint the boundaries between dehydration and desiccation tolerance. In this study, we subjected plants to gradual drying and quantitated their vitality upon rapid drying. Subsequently, we performed multiomics and network analyses to investigate the rate and mechanisms of desiccation tolerance in *T. loliiformis*. Our findings show that *T. loliiformis*, as a modified desiccation-tolerant plant, cannot survive rapid dehydration. Instead, gradual dehydration induces the accumulation of stress-associated metabolites, transcripts and phytohormones that suppress cell death. Like other resurrection plants, we propose that *T. loliiformis* undergoes a priming process during the early stages of dehydration, triggering the accumulation of specific transcripts, sugars, organic and amino acids by ca. 60% RWC to prepare the plant to not only tolerate desiccation but also fully recover once rehydrated. Despite rapid dehydration in *T. loliiformis* after 60% RWC, the initial gradual dehydration establishes the necessary mechanisms for desiccation tolerance. Root energy content at ca. 60% RWC coupled with the accumulation of salicylic acid and reduced jasmonate levels strongly suggest the importance of this RWC stage in attaining desiccation tolerance.

### Inducible protection mechanisms in modified desiccation tolerant plants

The cost of utilizing constitutive protective mechanisms decreases metabolic rates in truly desiccation tolerant plants compared to sensitive plants. As vascular plants evolved to possess larger and more complex physiology and structure, they lost their desiccation tolerance^43^. Consequently, vascular plants modified their tolerance mechanisms. Therefore, it is not surprising that when hydrated and mildly hydrated *T. loliiformis* plants, a vascular plant, were subjected to rapid dehydration, they showed a higher cell death rate, as observed in cell viability studies. Gradual dehydration provides *T. loliiformis* with sufficient time to employ costly tolerance strategies, such as water conservation mechanisms, while maintaining carbon fixation, slowing growth rate, and undergoing changes in structural and morphological properties that compromised their ability to survive rapid desiccation. Once *T. loliiformis* plants achieve desiccation tolerance, they can survive rapid dehydration, as seen by the rapid drop in RWC after ca. 60% shown in previous studies^8,11^.

Inducible cellular protective mechanisms play a significant role in modified desiccation-tolerant plants in acquiring tolerance^3,37^. Among these mechanisms, sugars and amino acids are crucial for plant responses to environmental stress^44–46^. During early dehydration, RFOs (raffinose family oligosaccharides), glucose, fructose and sucrose levels accumulate in plants^14,47^. The initial response of these sugar profiles in rice and Arabidopsis demonstrates a finely tuned metabolic response to dehydration^45^. Similarly, the early accumulation of sucrose, myo-inositol, and raffinose in *T. loliiformis* during dehydration potentially enables it to reprogram its metabolic pathways and prepare for desiccation. Amino acids such as proline, an important osmotic regulator, accumulate in various plants during drought, serving as osmolytes, signalling molecule and antioxidants^46^. Water deficit increases the use of branched chain amino acids as alternative pathways of respiration and their increased catabolism, rather than accumulation, has been linked to drought tolerance in Arabidopsis^29^. Elevated levels of aromatic amino acids, on the other hand, have been associated with secondary metabolite production^48,49^. Aromatic amino acids such as tryptophan, tyrosine and phenylalanine have shown an earlier response to water-deficit in Arabidopsis and rice^14,47,49^.

### Gradual drying and the early shutdown of photosynthesis allows *T. loliiformis* to regulate cellular energy status for desiccation tolerance by 60% RWC

The regulation of source/sink relationships is crucial in plant responses to environmental stresses and is dependent on strength and duration of the stress^50,51^. For example, during short term water deficit, Arabidopsis plants translocate sugars to the source (younger leaves) to maximize on energy production^50,51^. In contrast, during long term drought, Arabidopsis plants translocate carbon/sugars to the sink (roots), thereby promoting increased water uptake^51^. Like sensitive plants, when faced with long term water deficit, *T. loliiformis* sends its energy reserves to the roots. Dehydrated roots maintain high energy status by accumulating sucrose, T6P and T6P/SUC. These energy resources suppress snRK1 activation and autophagy ensuring that the roots are protected from the adverse effects of water deficit^10^. Due to this translocation, *T. loliiformis* plants survive gradual dehydration as seen by their recovery upon watering. Energy balance in the roots is maintained due to transport of carbon resources that provide necessary resources for continual water uptake from the soil. This slows down dehydration allowing the grass to employ additional physiological responses needed for desiccation tolerance.

The role of organic acids such as succinic acid, pyruvic acid, malic acid is not fully understood but they may be linked to disruptions in the TCA cycle during drought. Under moderate water deficit conditions, the TCA cycle remains unaltered^52^. However, in Arabidopsis, the TCA intermediates increased due to metabolic reprogramming triggered by drought^29^. Despite an increase in succinate, fumarate and malate, the responses of TCA intermediates in Arabidopsis vary^14,29,47^. Similarly, the accumulation of amino acids in *T. loliiformis* including tryptophan, tyrosine, proline, Isoleucine, tyrosine and phenylalanine during initial dehydration, coupled with lower activity in the TCA metabolites and the early shutdown of photosynthesis provide protection for dehydrating plants^8,11,53^. It then switches to an alternative respiration pathway once dehydration is perceived and fine-tunes the amino acid responses early enough to favour survival at 60% RWC, ensuring cellular protection and full recovery once favourable conditions are restored.

### *Tripogon loliiformis* manipulates jasmonate, salicylic and abscissic acid pathways to regulate cell death pathways and tolerate desiccation

Phytohormones play a significant role as signalling molecules that mediate growth, nutrient allocation and source/sink transitions during development and in response to environmental stresses^54^. Abscisic acid and salicylic acid are key phytohormones that regulate of abiotic stress signalling pathways^5,55–58^. Resurrection plants also utilize ABA to attain desiccation tolerance. For example, exogenous treatment of *C. plantagineum* plants with ABA induces dehydration-associated genes^24,37,59,60^. ABA also acts as an ROS scavenger^25^ and coupled with energy deprivation activate the SnRK1 metabolic sensor^38,61–64^. During stress periods, ABA levels increase in Arabidopsis plants which may lead to activation of snRK1 complex^38,65^. SnRK1 is activated during stress periods and activates autophagy which plays a role in remobilising nutrients that fuel stress responses, thereby contributing to stress tolerance^66^. Previously, we showed that *T. loliiformis* accumulates stress-associated metabolites in shoots and roots to suppress cell death^10^. We also established that shoots and roots elicit differential responses with the root acting as a sink during prolonged water deficit. We therefore propose that energy deprived *T. loliiformis* shoots use ABA signalling pathways to activate snRK1 and trigger autophagy. The presence of ABA in the hydrated state is not surprising because resurrection plants are primed to respond to water deprivation even in the hydrated state^67^. ABA may not be required by *T. loliiformis* shoots for desiccation tolerance but potentially for priming before onset of desiccation. Apart from ABA, accumulation of SA at 60% RWC potentially aids in attaining desiccation tolerance in *T. loliiformis*. Studies on *Phillyrea angustifolia* show that drought stress increases the endogenous levels of SA by five-fold^68^ while barley roots recorded two-fold increases in SA upon water deficit^69^ confirming that SA plays a role in plant responses to drought stress.

Jasmonates (JAs) activate apoptotic cell death in Arabidopsis protoplasts^70^. They also induce the production of reactive oxygen species when synthesized in response to stress^71,72^. Treatment of Arabidopsis and tobacco suspension-cultured cells with methyl jasmonate (MeJa) induces an ROS burst^73,74^. While these observations suggest a potential role of JAs in promoting cell death, some studies have linked JAs to drought tolerance^75,76^. Increased levels of JA have been observed in rice shoots and maize roots during drought stress^77,78^. Jasmonates and particularly MeJa induce apoptosis in mammalian cancer cells^79,80^. Given that jasmonates can induce apoptosis via ROS bursts, the reduction of jasmonates in *T. loliiformis* during dehydration could aid in suppressing apoptosis during dehydration. Jasmonates induce apoptotic-like cell death in Arabidopsis protoplasts via the fungal toxin fumonisin B1^81^. Additional studies in human cancer cell lines showed that jasmonates induced apoptosis in a caspase 3 -dependent manner that increases expression of Bcl^−^2 and Bax apoptotic proteins via ROS signalling pathways^82,83^. Jasmonates play a role in cell cycle development. MeJa arrests cell cycle at distinct phases in mammalian cancer cells^70,84,85^. The higher JA concentrations during mild dehydration in *T. loliiformis* could be attributed to the plant's cell cycle while undergoing growth and development.

In summary, most plants, including angiosperms, require sufficient water for proper growth and development. Reducing RWC to 60% is very harmful to plants. It is at this critical point that plants determine their response by exhibiting drought escape, avoidance, or drought-tolerance strategies^86^. *T. loliiformis* is unable to survive rapid desiccation and therefore initiates dehydration-induced tolerance mechanisms. Gradual drying triggers signalling of stress-associated TFs, regulation of the transcriptome to maximize on carbohydrate and energy metabolism, antioxidant production and the accumulation of stress-associated metabolites and phytohormones that enable the plant to mitigate periods of prolonged water deficit. At 60% RWC, *T. loliiformis* plants send their energy reserves to the roots protecting the roots from adverse effects of prolonged drought. The lower energy levels in *T. loliiformis* shoots following carbon translocation to the roots cause the early shutdown of photosynthesis and metabolism preventing water loss and activating protective pathways. Additionally, lower energy levels in shoots trigger activation of other physiological and metabolic responses needed for desiccation tolerance. In doing so, the grass accumulates sugars, amino acids, SA, decrease JAs accumulation and initiate autophagy through snRK1 activation at 60% RWC, avoiding apoptosis, increasing nutrient remobilization, and maintaining cellular homeostasis. While both resurrection and sensitive plants respond similarly to initial water deficit, prolonged drought causes a stark difference in the responses. Sensitive plants initiate drought escape mechanisms by induction of senescence and flowering. *T. loliiformis,* like most resurrection plants, employs more complex mechanisms, including autophagy to tolerate drought^10,11^. An understanding of the desiccation tolerance mechanisms utilized by resilient plants like *T. loliiformis* provides knowledge that will be useful in the improvement of yield in crops of economic importance through the generation of stress-tolerant plants.

## Materials and methods

### Plant materials and cultivation

*Tripogon loliiformis (F.Muell.) C.E.Hubb.* plants were previously collected (permit # WISP09847111) in 2012 from Charleville (GPS: − 26.42686 S, 146.25002E), Queensland, Australia. One inbred line derived from a single mother plant was used for all the experiments (Queensland Herbarium voucher accession number: Williams01). *T. loliiformis* plants were germinated from seeds collected from a single plant and grown in a chamber at 27 °C and 16 h photoperiod. Twenty-one, 65 mm pots, each containing multiple plants were grown for two months and watered to saturation. Hydrated controls (FH) were randomly collected in three replicates, one-day post-watering. Water was withheld from the remaining plants until they were desiccated (air dry) and their relative water content (RWC) dropped below 10% (dehydrated, DH). Triplicate samples for metabolomic analysis were collected once the plants were at a RWC of 100–80%, 55–65%, 35–45% and < 10% (DH). Shoot samples were taken and immediately snap frozen in liquid nitrogen until processed. For collection of root samples, plants were uprooted, and the soil was removed by washing under water for five to ten seconds before patting dry using paper towel and snap freezing in liquid nitrogen. The plants were desiccated approximately ten days post-termination of watering. Rehydrated (RH) samples were collected 48 h after watering. The percentage RWC was determined on *T. loliiformis* shoots and roots and was calculated according to Barrs and Weatherley^87^ using the formula (RWC (%) = ((Fresh Weight − Dry Weight)/(Turgid Weight − Dry Weight)) × 100).

### Dehydration assessment

*T. loliiformis* plants were gradually dried to different dehydration states (100–80, 70–60, 50–40, 30–20 and < 10% RWC) before uprooting from the soil and rapidly desiccating in an oven (30 °C) for 20 h until their RWC became < 10% before returning to the soil. Hydrated plants were used as controls. For each dehydration point, five replicates were used. The plants were re-planted in the soil, watered, and observations were made after 72 h. Data was collected on the number of plants uprooted, the number of plants that died, and the number of plants that resurrected after rehydration.

### Cell viability assay using Evans Blue

Whole* T*. *loliiformis* plants were harvested from replanted and rehydrated plants, placed into 2 mL microfuge tubes, and soaked in water for 2 h to facilitate staining. Hydrated leaves were boiled for 5 min and served as controls for positive Evans Blue staining. Each plant was submerged in 0.25% Evans Blue stain and incubated at room temperature for 20 min before washing with distilled water three times. Stained cells were visually assessed by light microscopy before spectroscopy for quantitative analysis. The plants were ground to a fine powder and incubated at 37 °C with 1% sodium dodecyl sulphate (SDS) for 10 min. The samples were clarified by centrifugation at 14,000 rcf for 10 min. The supernatant was transferred to a fresh tube. The absorbance of each sample was measured at 600 nm. Samples at ca. 80% RWC, ca. 60% RWC, ca. 40% RWC and < 10% RWC were selected as representatives for further analysis.

### RNA-seq analysis

Previously generated data^10,11^ were used. Using hydrated samples as a reference, each data set (one data set per RWC percentage) was enriched for genes that had an absolute fold change of ≥ 2 and an adjusted (Benjamini Hochberg) *p* value < 0.05. Transcription factors, drought stress-related genes and genes associated with energy metabolism, signalling and antioxidation were identified. Next, transcript data derived from roots were separated from those derived from shoots. The top 10,000 genes (ranked by coefficient of variation, CV) were pulled from each data set. Pearson correlation coefficients were calculated for each gene pair in these sets. We then inferred a network using this Pearson data from roots and a separate network for the data from shoots. For both networks, any gene pair with an absolute value of Pearson correlation coefficient > 0.964 was included. Networks were viewed and analysed using Cytoscape^88^.

### GC–MS analysis of metabolites in hydrated, dehydrating and desiccated *T. loliiformis* shoots and roots

To analyse changes in *T. loliiformis* metabolite accumulation during dehydration, gas chromatography-mass spectrometry (GC–MS) based metabolomics analysis was performed. Two-month-old hydrated, dehydrating (ca. 80, ca. 60 & ca. 40% RWC), dehydrated (< 10%) and rehydrated plants were harvested, snap-frozen in liquid nitrogen and lyophilised overnight. Following lyophilization, the dry weight was measured for normalisation and the samples were ground to a powder using a Qiagen tissue lyser (2 × 1 min). Metabolites were extracted using water, chloroform and methanol^89^ and the top layer of methanol/water which contains polar metabolites was analysed by GC–MS. Metabolites were initially identified by matching experimental spectra to an augmented version of the Agilent Fiehn Metabolomics Library, containing spectra and validated retention indices for almost 1000 metabolites^90^ and additionally cross-checked by matching with NIST20 GC/MS Spectral Library and Wiley Registry 11th edition. Metabolite identification was confirmed by the mass spectral similarity of fragmented spectra and closeness of retention index values calculated based on the separation of fatty acid methyl esters (C8–C28). All metabolite identifications were manually validated to minimize deconvolution and identification errors during the automated data processing. All experiments were conducted using three biological replicates.

### LC–MS analysis of phytohormone levels in hydrated, dehydrated-states and desiccated *T. loliiformis* shoots and roots

To analyse changes in *T. loliiformis* phytohormone accumulation, specifically abscisic acid (ABA), salicylic acid (SA) and jasmonic acid (JA) during dehydration, LC–MS analysis was performed. Two-month-old hydrated, dehydrating (80, 60 & 40% RWC) and rehydrated plants were harvested, snap-frozen in liquid nitrogen and lyophilised overnight. Following lyophilization, the dry weight was measured for normalisation and the samples were ground to a powder using a Qiagen tissue lyser (2 × 1 min). For each sample, 100 mg frozen roots were weighed into a 2 mL Lysing Matrix D tube (MP Biomedicals, USA). The Lysing Matrix D tube was prewashed using 70% methanol. Subsequently, 1 mL 70% methanol containing 5 μL internal standard (ISTD) working solution (500 ng mL^−1^ salicylic-d_6_ acid, 100 ng mL^−1^ jasmonic acid and 20 ng mL^−1^ d_6_-2-*cis*-4-*trans*-ABA) was added to the sample. Samples were then homogenized using a Cryomill coupled to a Cryolys cooler (Bertin Technologies, France) set to -10 °C (6,800 rpm, 3 × 30 s, 30 s break) followed by shaking for 30 min at 900 rpm at 4 °C. Then, samples were centrifuged at 15,900 rcf at 4 °C for 5 min. The supernatant was transferred to a 2 mL Eppendorf tube and dried using a rotational vacuum concentrator (Christ, Germany) under full vacuum at 30 °C. After that, the dried extract was reconstituted in 50 μL of starting mobile phase [5% acetonitrile (ACN) with 10 mM ammonium acetate (NH_4_Ac)] and subsequently sonicated for 10 min until the dried extract dissolved. The extract was centrifuged at 15,900 rcf at 4 °C for 15 min prior to transfer to an amber vial with glass insert. Samples were stored at − 80 °C until LC–MS analysis. Standard stock solutions were prepared at 50 μg mL^−1^ and working solutions at 1 μg mL^−1^ in methanol. All stock solutions and working solutions were stored at − 80 °C. The LC–MS system was an Agilent 1290 series high performance liquid chromatograph. Phytohormones were separated on a Phenomenex Kinetex C18 reversed phase column (2.1 mm × 100 mm, 1.7 μm) maintained at 45 °C. The mobile phases and gradient were as follows: mobile phase A: 10 mM NH_4_Ac in deionized water; mobile phase B: 10 mM NH_4_Ac in ACN. The mass spectrophotometry data was annotated using a pre-existing mass spectra repository and databases as described by Dias et al.^91^. Electrospray ionisation of mass spectra was recorded at a scanning range of 30–650 m/z. All experiments were conducted using three biological replicates.

### Statistical analyses

Data on resurrected plants, cell viability, GC–MS analysis was collected and analysed using Minitab software Version 17. ANOVA was conducted to determine the significance at *p* < 0.05.

All the methods were carried out in accordance with Queensland University of Technology guidelines and regulations.

### Supplementary Information

Supplementary Table S1.

Supplementary Table S2.

## Data Availability

The transcriptome data sets analysed during this study are deposited and available from the Sequence Read Archive (SRA) at NCBI, Accession number PRJNA288839. The proteomic dataset generated and analysed is available upon request from the corresponding author (b.williams@qut.edu.au).
