# Supplementary material for: Tripogon loliiformis tolerates rapid desiccation after metabolic and transcriptional priming during initial drying
Source: Sci Rep. 2023 Nov 23;13:20613. doi: 10.1038/s41598-023-47456-3 (PMC10667271; doi:10.1038/s41598-023-47456-3)
Supplement: Supplementary file 1 — Supplementary Table S1. [file 41598_2023_47456_MOESM1_ESM.doc]

**STable 1:** DEGs upregulated (Green) and downregulated (Red) and their position in the shoot and root network analysis. P<0.05 and FDR 5%.
